# An Examination of the Low Strain Rate Sensitivity of Additively Manufactured Polymer, Composite and Metallic Honeycomb Structures

**DOI:** 10.3390/ma12203455

**Published:** 2019-10-22

**Authors:** Quoc Lam, Dhiraj Patil, Thao Le, Trevor Eppley, Ziyad Salti, Derek Goss, Alex Grishin, Dhruv Bhate

**Affiliations:** 1 The Polytechnic School, Arizona State University, Mesa, AZ 85212, USA; quocl2013@gmail.com (Q.L.); dapatil@asu.edu (D.P.); ms21194@yahoo.com (T.L.); sparkyeppley@gmail.com (T.E.); ziyadzsalti@gmail.com (Z.S.); dlgoss@asu.edu (D.G.); 2 Phoenix Analysis & Design Technologies Inc., Tempe, AZ 85284, USA; alex.grishin@padtinc.com

**Keywords:** honeycomb, additive manufacturing, 3D printing, cellular, lattice, foam, strain rate, viscoplastic

## Abstract

The characterization of additively manufactured cellular materials, such as honeycombs and lattices, is crucial to enabling their implementation in functional parts. One of the characterization methods commonly employed is mechanical testing under compression. This work focuses specifically on the dependence of these tests to the applied strain rate during the test over low strain rate regimes (considered here as 10^−6^ to 10^−1^ s^−1^). The paper is limited to the study of strain the rate dependence of hexagonal honeycomb structures manufactured with four different additive manufacturing processes: one polymer (fused deposition modeling, or material extrusion with ABS), one composite (nylon and continuous carbon fiber extrusion) and two metallic (laser powder bed fusion of Inconel 718 and electron beam melting of Ti6Al4V). The strain rate sensitivities of the effective elastic moduli, and the peak loads for all four processes were compared. Results show significant sensitivity to strain rate in the polymer and composite process for both these metrics, and mild sensitivity for the metallic honeycombs for the peak load. This study has implications for the characterization and modeling of all mechanical cellular materials and makes the case for evaluation and if appropriate, inclusion, of strain rate effects in all cellular material modeling.

## 1. Introduction

Cellular materials such as honeycombs and lattices, represent some of the most exciting design frontiers in additive manufacturing (AM). While these materials have been used in several engineering applications and are the focus of at least three books [1,2,3], developments in AM process technologies and software tools have led to a resurgence of interest in them. This is primarily due to the greater freedom to not only design and manufacture cellular materials that were hitherto not feasible, but also due to the ability to seamlessly integrate these materials into three-dimensional structures.

While the potential for cellular materials made with AM in engineering applications is large, these materials have only found widespread end-application in biomedical implants, where porosity provides significant advantages [4]. The aerospace industry, while being an early adopter of AM technology, has few examples of cellular material designs implemented in load-bearing applications, with most examples being related to part consolidation and topology optimization, or to address lead time reductions and supply chain complexities [5]. A key reason for this is the uncertainty associated with the performance of cellular materials, which in turn is traced to uncertainty introduced by the manufacturing process, the design and the materials used, and to the experimental and computational methods used to develop predictive models [6,7]. 

When developing a model that is predictive of cellular material behavior, the primary metrics of interest are in elastic properties, such as elastic modulus, and some description of the nonlinearity that follows, typically in terms of a failure stress. One aspect that can influence the computation of these properties is the strain rate, and that is the focus of the present work. It is typically assumed that strain rates between 10^−2^ and 10^−3^ s^−1^ may be considered for testing cellular materials, following recommendations in the ISO 13314 standard [8]. There is a substantial body of work on the dynamic, compressive behavior of cellular materials, most recently reviewed by Sun and Li [9], who examined the literature of strain rates in three regimes: quasi-static, transitional-dynamic and shock, focusing on explaining mechanisms and the possible discrepancies in the data, particularly at high strain rates. Gumruk et al. showed strain rate sensitivities for AM stainless steel struts, but did not examine strain rates between 10^−5^ and 1 s^−1^ [10]. 

Compressive load studies on AM cellular materials have been conducted by the prescription of velocities instead of strain rates, often set to 5 mm/min, a value that originates from the standard for tensile testing of bulk plastics and approximates to 10^−3^ s^−1^ for most cellular structures tested [11,12]. Other studies have been conducted at a range of velocities, including 0.4 mm/min [13], 1 mm/min [14] and 1 mm/s [15], or at strain rates of 10^−3^ s^−1^ [16], to cite a few examples. In addition, most of these studies use their findings to compare results across different cellular material designs, implicitly assuming equivalence of strain rates. 

It is well known that cellular material, such as foams, respond differently under shock/impact relative to a lower strain rate [9,17,18]. This is on account of dynamic factors, such as micro and macro-inertia, and wave effects at high strain rates that are not relevant at lower rates [9]. The focus of this paper was, however, at the very low strain rate regime, at and below the 10^−2^ s^−1^ recommended standard by ISO. At these low strain rates, rate dependence can originate from two sources: the rate dependent properties of the base material itself, and the contribution of the structural heterogeneity inherent in cellular materials to the local strain rates. Each of these is discussed and demonstrated in turn below. 

**Figure 1:**
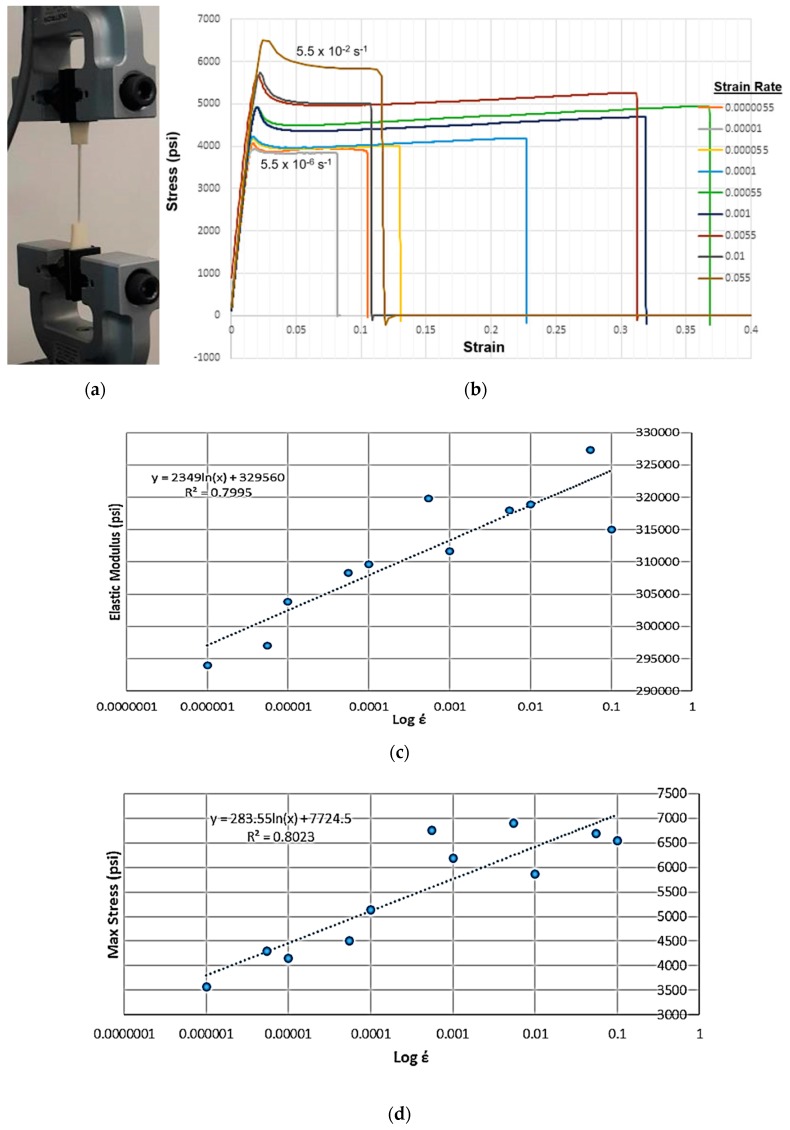
(**a**) Test setup used to establish material properties for ABS on thin specimens [6]. (**b**) Strain rate dependent mechanical behavior recorded for these specimens. (**c**) Semi-log plot showing the rate dependence of both elastic moduli, and (**d**) the peak stress values measured during these tests.

One source of rate dependence in cellular materials stems from the viscoplasticity of the base material itself, such as ABS [19], which like most plastics, is known to be strongly viscoplastic (rate dependent), even at room temperature. Figure 1 shows the results of a study on thin dog-bone specimens that were fabricated with the fused deposition modeling (FDM) process to mimic the walls of the hexagonal honeycomb. These specimens were tested on a 1kN INSTRON tensile tester, as shown in Figure 1a; information on the experimental setup can be found in [6]. This specimen was pulled at strain rates ranging from 5.5 × 10^−6^ s^−1^ to 5.5 × 10^−2^ s^−1^, the latter corresponding to the fastest velocity the crosshead was able to travel at. Figure 1b shows the experimental response, showing strain rate dependence. Figure 1c,d are semi-log plots showing the dependence of elastic modulus and the peak (maximum) stress as a function of strain rate, showing a robust linear correlation with *R^2^* values of approximately 0.8. 

**Figure 2:**
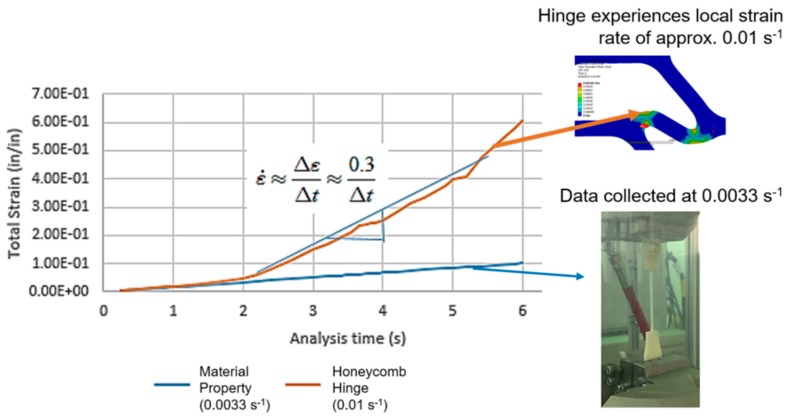
Simulation of a honeycomb under compression shows that local strain rates within the cellular material can be significantly larger than those assumed in the material model (derived from the testing of a bulk specimen) and applied in the test.

Rate dependence of the base material is a well-known phenomenon; however, what is less understood is the role of geometry in creating a state of heterogeneous strain, and by extension, heterogeneous strain rate through the cellular material. This can be demonstrated through studying the results of a finite element analysis (FEA) simulation, where a compression load was applied to a hexagonal honeycomb with quasi-static isotropic material properties from previously-mentioned ABS dog-bone specimens (Figure 2). The same effective strain rate was applied to the hexagonal honeycomb (using the height of the honeycomb as its effective gauge length). As the simulation demonstrates, the accumulation of strain in the diagonal beams (consistent with the plastic hinge reported by Gibson and Ashby [1]) occurs at a faster rate (approximately 0.01 s^−1^) than that experienced by the specimen whose material properties were used as an input material property. In other words, a globally applied strain rate on a cellular material can generate local strain rates that can be an order of magnitude higher. 

There are two implications of this brief study: The first is that one cannot assume that the applied strain rate on a cellular material under compression is sufficient in describing behavior occurring within the structure. The second is that local strain accumulation rates can influence overall, global behavior—as such, it is not evident that all geometries will behave the same, since the accumulation of strain is a function of local geometry.

This paper examines the strain rate dependence of cellular materials at low strain rates, spanning a range of 10^−6^ to 1 s^−1^, and in so doing questions the assumption of quasi-static behavior of cellular materials at these strain rates. To simplify this study, the work was limited to the compression of prismatic cellular materials, specifically hexagonal honeycombs, but examined behavior in structures manufactured with four different AM processes: fused deposition modeling (FDM) with ABS; material extrusion with a nylon-carbon fiber and continuous carbon fiber blend; electron beam melting (EBM) with Ti6Al4V; and finally, laser powder bed fusion (L-PBF) with Inconel 718. Findings were compared across different processes, and the paper concludes with a discussion of the implications of these findings for future work. 

## 2. Materials and Methods 

**Figure 3:**
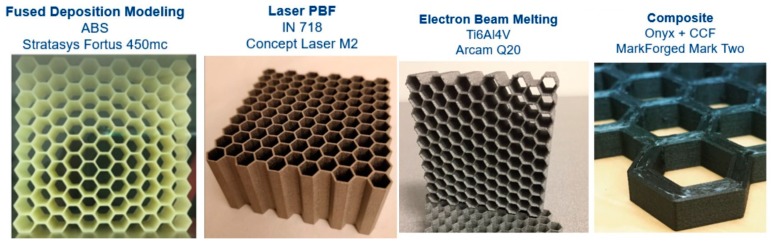
Honeycomb specimens fabricated on four different processes, with the same number of cells and 1 in thicknesses, but with varying cell sizes and wall thicknesses. The composite honeycomb had continuous carbon fiber (CCF) with an Onyx^TM^ (nylon + chopped carbon fiber) matrix.

To better understand the role of strain rate sensitivity at the low to intermediate strain rates, hexagonal honeycombs were manufactured from four different AM processes, all designed in accordance with ISO 13314 [8] and previous size effect studies [12,20,21], as shown in Figure 3 and summarized in Table 1. A minimum of 10 cells formed the honeycomb array, with an extrusion thickness of 1.0 in. The only exception was the composite honeycomb, which only had four cells due to contour thickness limitations and the build envelope of the machine. Build direction for all samples was perpendicular to the honeycomb arrays. Cell dimensions, such as cell wall thickness and size varied between each set of materials, and is discussed further in the following sub-sections.

Tests were conducted on an INSTRON 8801 servo-hydraulic mechanical tester (Norwood, MA, USA) with a 50 kN load cell under compression at varying effective strain rates, nominally specified as displacement rates with the height of the honeycomb used as an effective gauge length to estimate effective strain. 

### 2.1. Fused Deposition Modeling with ABS 

**Figure 4:**
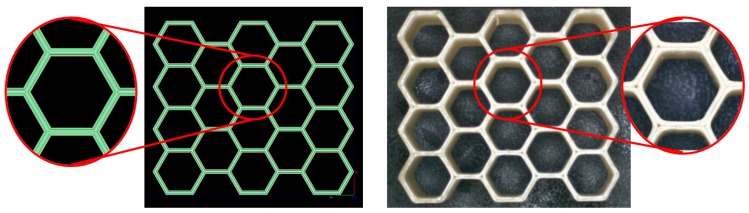
FDM toolpaths and evaluation print for honeycomb structures (this image shows a sample honeycomb smaller in size than the actual honeycomb tested for the data reported here). Two contours of 0.016 “were fitted within the 0.032” wall thickness.

The FDM honeycombs were manufactured on the Stratasys Fortus 450mc machine with the ABS-M30 material by Stratasys (ivory color). To minimize printing process-induced variation, samples were created in three separate, but successive builds on the same machine and tipped with no re-calibrations performed between builds. Printing parameters were set as: layer thickness = 0.010 in and contour width = 0.016 in. For a wall thickness of 0.032 in, completed parts were mechanically removed, without further post-processing. An example of Stratasys’s Insight^TM^ software’s tool path and a corresponding sample print is shown in Figure 4. 

### 2.2. Composite Material Extrusion with Nylon-CF Composite and Continuous CF 

**Figure 5:**
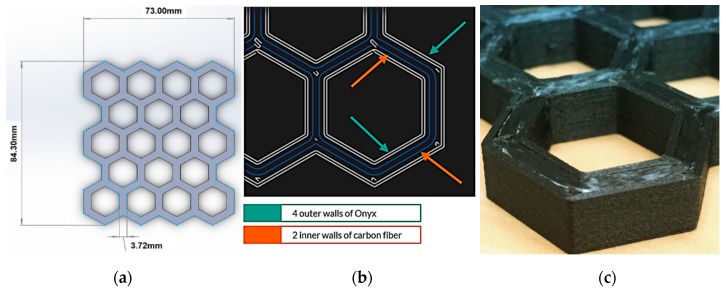
(**a**) Design which needed to be have fewer cells than desired due to size limitations; (**b**) material layout as specified in the build preparation software; and (**c**) fabricated part (paused in mid-print), showing the distribution of continuous carbon fiber and Onyx.

Composite material extrusion was enabled using a MarkForged Mark Two 3D printer (Watertown, MA, USA), which utilizes two nozzles: one dedicated to Onyx^TM^, a nylon matrix with chopped carbon fiber; the second, continuous carbon fiber (CCF). Both materials were extruded to create a honeycomb with two contours of CCF embedded within two contours of Onyx, as shown in Figure 5. Parameters were selected to generate the thinnest possible walls that would result in a symmetrical distribution of fiber throughout the structure, and in effect, limited cell count was compared to other processes. This is because the smallest size of wall that could accommodate the required minimum number of Onyx and CCF contours were 0.146 in, which in turn drove the need for very large cells sizes in order to resolve the cells and generate consistent prints.

### 2.3. Electron Beam Melting with Ti6Al4V

**Figure 6:**
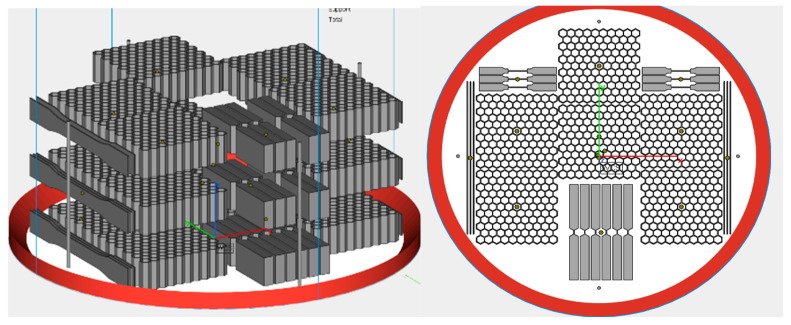
(**Left**) Build layout for the hexagonal honeycomb specimens, as manufactured in the Arcam Q20 EBM system. (**Right**) Top view.

Arcam’s Q20 machine was selected for the EBM process, using Ti6Al4V powder material. Since the samples were fabricated at a partner facility using their proprietary parameters and support designs, those details cannot be reported here. All test samples were nested within one build, as shown in Figure 6. 

### 2.4. Laser Powder Bed Fusion with Inconel 718

Inconel 718 was selected as the material of interest and was manufactured using a Concept Laser M2 (400W) machine. As with the EBM samples, print parameters must remain undisclosed as a result of proprietary knowledge of the independent vendor; however, a 40 micron layer thickness was requested and used. Following build completion, samples underwent supplier-recommended stress-relief solution annealing and aging prior to testing. 

## 3. Results

### 3.1. Fused Deposition Modeling with ABS

**Figure 7:**
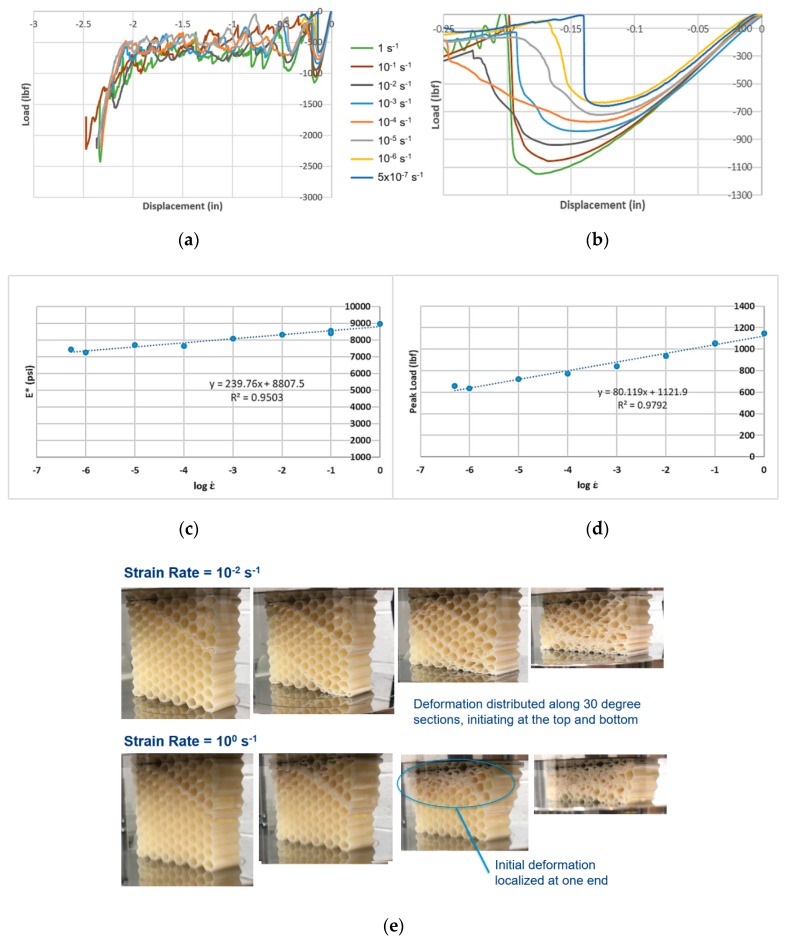
(**a**) Load-displacement graphs for FDM ABS hexagonal honeycomb across seven orders of effective strain rate magnitude. (**b**) Magnified version of the same graph. (**c**) Effective modulus E*–effective strain rate semi-log plot. (**d**) Peak load–effective strain rate semi-log plot. (**e**) Deformation patterns at two strain rates: at 1 s^−1^, deformation tends to get localized at one end (lower platen is moving up).

Compression test results for the FDM ABS honeycombs are shown in Figure 7a,b, focusing on the initial collapse of an array, showing how the peak load rises with increasing effective strain rate. As the measured peak load and effective elastic modulus, E*, are plotted over their respective strain rates, a robust linear correlation is visible (Figure 7c,d). As strain rates increase, deformation patterns begin transitioning from the “homogeneous” model of deformation to the “transition” mode, prior to reaching a “shock” mode, where deformation is highly localized at the shock front (Figure 7e) [22]. 

### 3.2. Composite Material Extrusion with Nylon-CF Composite and Continuous CF

**Figure 8:**
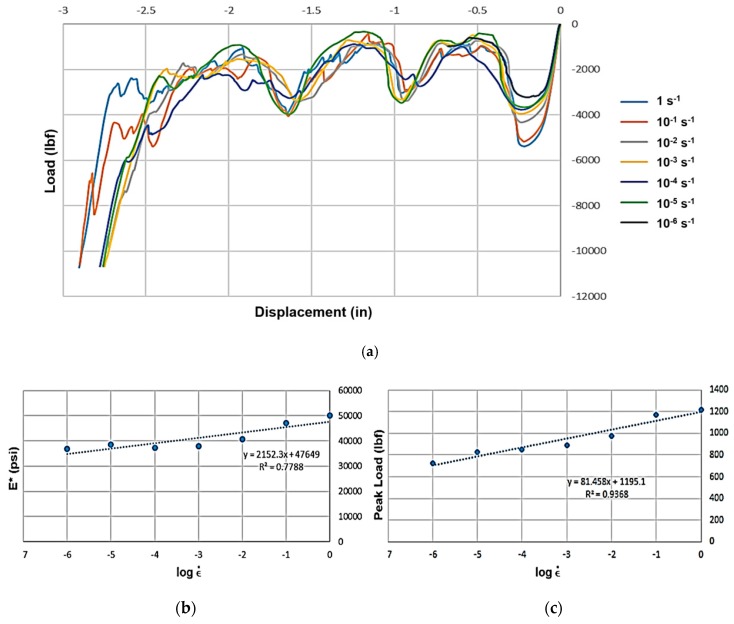
(**a**) Load-displacement graphs for composite hexagonal honeycomb across seven orders of effective strain rate magnitude. (**b**) Effective modulus E*–effective strain rate semi-log plot; (**c**) peak load–effective strain rate semi-log plot.

While the composite honeycombs have a number of cells that is smaller than desired, the ability to query strain rate dependence is not hindered. It does, however, imply the reported moduli and peak loads are under-predicted, as is commonly the case for cellular materials tested in compression and consistent with prior observations for hexagonal honeycombs [12,21]. As such, modulus and peak load dependence on strain rate remained similar to those of ABS (Figure 8a), albeit, with slightly less correlation from each analysis with R^2^ equal to 0.7788 and 0.9368, for E* and peak load, respectively (Figure 8b,c). 

### 3.3. Electron Beam Melting with Ti6Al4V

**Figure 9:**
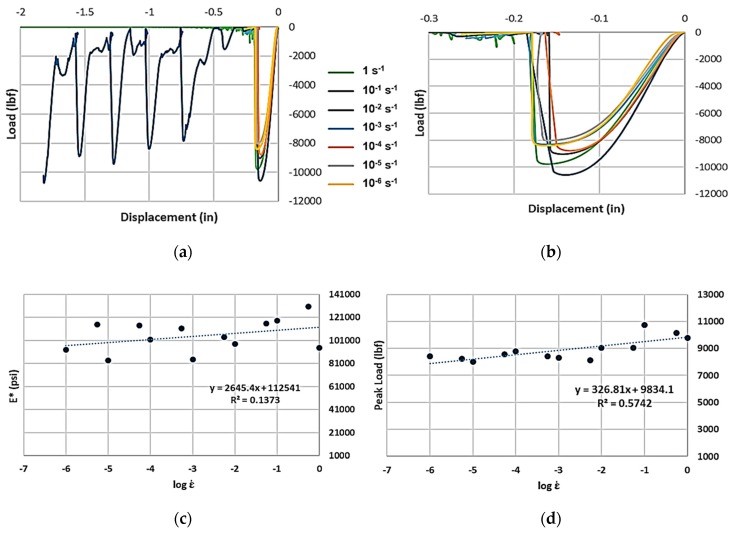
(**a**) Load-displacement graphs for EBM Ti6Al4V hexagonal honeycomb across seven orders of effective strain rate magnitude. (**b**) Magnified image of initial failure response showing sharp drop in load consistent with sudden, brittle failure. (**c**) Effective modulus E*–effective strain rate semi-log plot; (**d**) peak load–effective strain rate semi-log plot.

**Figure 10:**
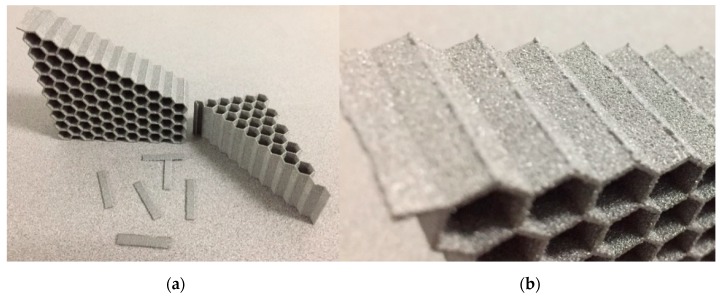
(**a**) Nodal failure and separation of EBM Ti6Al4V specimens without densification. (**b**) Failure occurred close to the corners, with no evident plastic hinging and clear separation of the walls from the honeycomb structure.

Unlike the polymer and composite specimens, strain rate dependence of the Ti6Al4V was not immediately evident, as shown in Figure 9a,b. Additional tests (not shown in Figure 9a,b) were conducted at interpolated strain rate values and the effective modulus and peak load plotted in Figure 9c,d show some strain rate dependence, though with lower correlations compared to the previous datasets. Additionally, observations during testing showed brittle failure near the corners (Figure 10a,b). No densification was observed (with the exception of one sample) since the specimen broke off into two pieces and the test was halted. In one case, the broken piece managed to slot itself into position and subsequent densification was observed, as can be seen for a single curve in Figure 9a. 

### 3.4. Laser Powder Bed Fusion with Inconel 718

**Figure 11:**
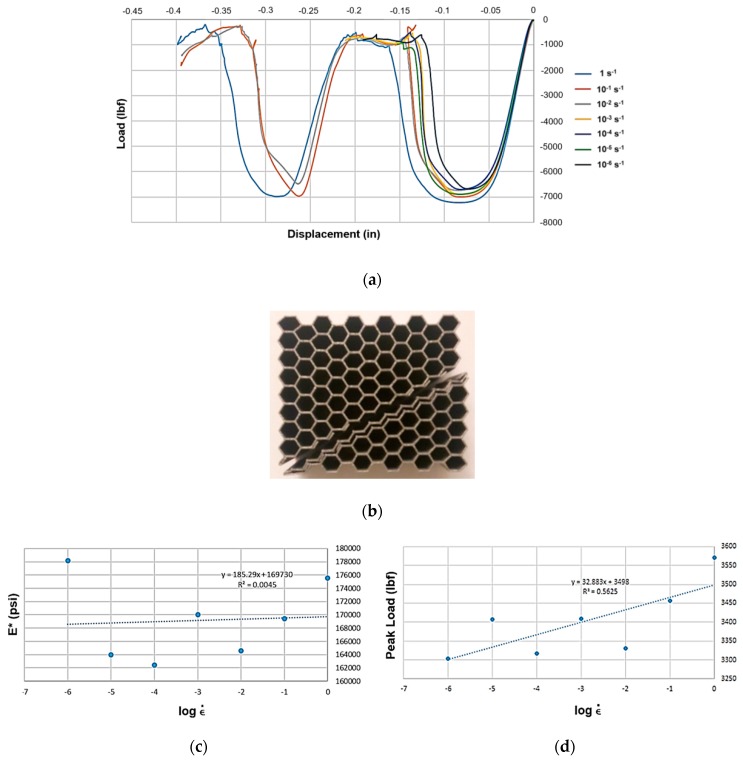
(**a**) Load-displacement graphs for LPBF Inconel 718 hexagonal honeycomb across seven orders of effective strain rate magnitude. (**b**) Post-failure honeycomb. (**c**) Effective modulus E*–effective strain rate semi-log plot; and (**d**) peak load–effective strain rate semi-log.

As shown in Figure 11a,b, the LPBF Inconel 718 honeycombs had a failure pattern closer to the EBM honeycombs shown previously, but for the three highest strain rates they did allow for two rows of cells to collapse prior to complete separation. As can be seen from Figure 11c, the effective modulus showed little influence of strain rate, but the peak load did show a moderate influence. 

### 3.5. Strain Rate Sensitivity

As suggested in the preceding results, not all processes and materials have the same strain rate response at the range of strain rates studied here. One method of comparing the strain rate sensitivity of the different processes and materials involved in this study is to express the effective elastic modulus and peak stress in terms of power law relationships:

(1) $$E^{*}=C{\dot{\epsilon }}^{m}$$

(2) $$\sigma^{*}=K{\dot{\epsilon }}^{n}$$

where C and K are multiplicative constants; m and n are exponents indicative of the strain rate sensitivity; i.e., the higher the exponent, the greater the sensitivity of the metric of interest to strain rate. While this is not the only way of modeling these relationships, it was selected based on its simplicity since the aim of this paper was to arrive at a metric of comparison for strain rate sensitivity and not propose a predictive model. 

**Figure 12:**
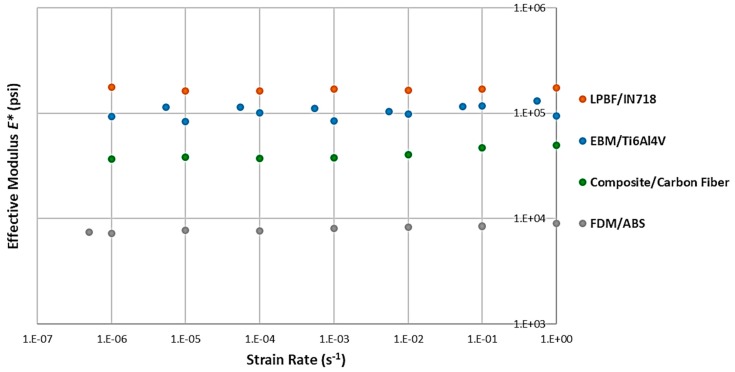
Strain rate sensitivity of the effective elastic modulus for each of the four different processes.

A –log plot was created for the four material’s (effective) elastic modulus (Figure 12). This data was then further fit into Equation (1), with the estimated strain rate sensitivity listed in Table 2, which clearly shows significant strain rate sensitivity for modulus for all processes except EBM. Additionally, the quality of the fit is reasonably high for the ABS and composite honeycombs, but significantly lower for the metallic samples, suggesting the need for further investigation. 

**Figure 13:**
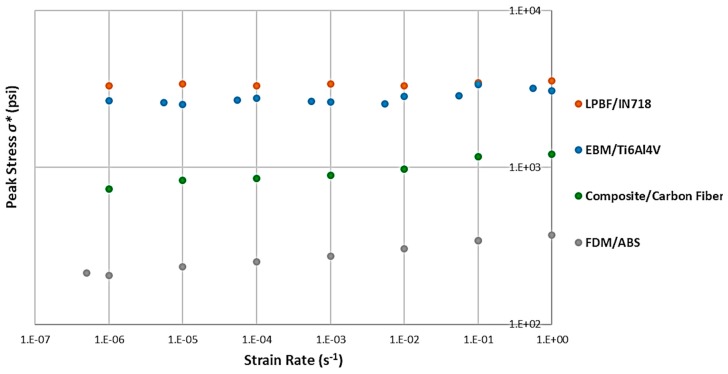
Strain rate sensitivity of the peak stress for each pf the four different processes.

The same approach was taken for effective peak stress (Figure 13 and Table 3). In this case, LPBF was the process that did not show a significant strain rate sensitivity for the peak stress, at a reasonably high correlation value. ABS and composite honeycombs showed quite high sensitivities, followed by Ti6Al4V with EBM.

## 4. Discussion

Several researchers have conducted tests on cellular materials at an effective strain rate value of about 10^−3^ s^−1^, but our findings suggest that this value may not be low enough for the quasi-static assumption to hold, especially in the case of polymeric cellular materials. For metals this effect is not significant at ambient temperatures, though some minor sensitivity is observed.

**Figure 14:**
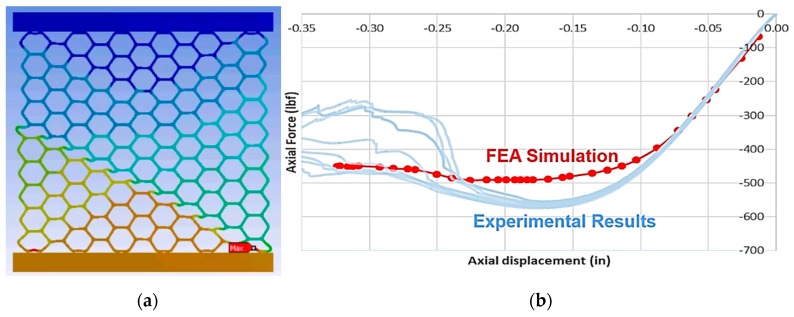
(**a**) 2D plane strain FEA model of hexagonal honeycomb under compression showing plastic hinge formation consistent with experimental observations, and (**b**) a comparison between FEA and experimental results showing good agreement in the elastic regime, but underprediction of plastic response.

There are at least two potential implications of not considering rate-dependent behavior when characterizing cellular materials. The first of these is the impact on our ability to develop predictive material models. Consider Figure 14a, which shows the results of a static, 2D, plane-strain finite element analysis (FEA) of a hexagonal honeycomb compared to the experimental results for a compression test on a honeycomb conducted at an effective strain rate of 10^−3^ s^−1^. The FEA material model had elastic plastic properties estimated using the test setup shown previously in Figure 1a, that were estimated at a strain rate of 3.3 × 10^−3^ s^−1^. Despite the fact that these strain rates were so close, there is still a significant under-prediction in the plastic response of the honeycomb, as shown in Figure 14b. This is likely to be on account of the lack of modeling-rate-dependent behavior in the FEA model.

The second potential implication has to do with using compression data to draw similarities between different cell shapes and occasionally different processes, as has been done in the past. It is not evident, for example, that the strain rate sensitivities for all cellular shapes are identical; in other words, even if two cellular shapes (a hexagonal honeycomb and an octet lattice, for example) are subjected to identical effective strain rates, they may have different strain rate sensitivities specific to each geometry and the manner in which strain accumulates locally. In such cases, it is recommended that initial testing at a few different strain rates be conducted to verify the comparisons qualitatively. More work is needed to study and incorporate strain rate dependence of cellular materials into the design and analysis process. 

**Figure 15:**
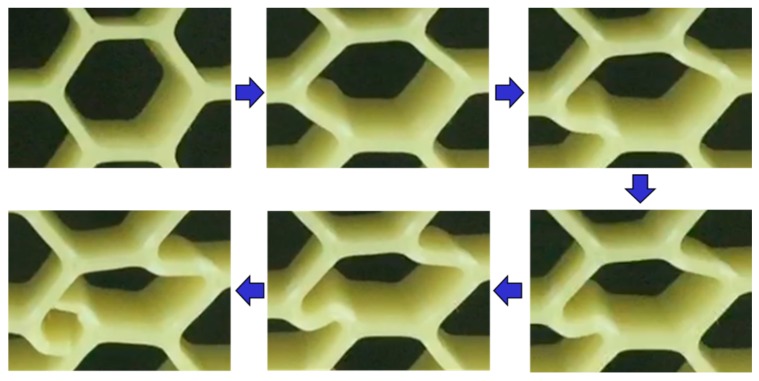
Formation of plastic hinge resulting from beam bending (shown here for ABS honeycombs made with the fused deposition modeling process).

While a detailed failure analysis is out-of-scope of this work, focused as it is on the strain rate dependence of the effective modulus and the peak load only, it is worth pointing out that failure in honeycombs is the end result of deformation induced through some combination of beam bending, and shear and axial loading [1]. For fused deposition modeling, for example, as shown in Figure 15, plastic hinging resulting from beam bending results in separation of the two contour beads and subsequent separation of the entire wall. In contrast, the failure mode of the EBM Ti6Al4V shows no beam bending and hinging (see Figure 10). These differences in failure modes may explain some of the differences in the dependencies in strain rate observed as well. 

## 5. Conclusions

This work examined the strain rate sensitivity for four different additively-manufactured honeycomb structures over the strain rate range from 10^−6^ to 10^−^^1^ s^−1^, at room temperature, and drew the following conclusions, applicable within that range:

- Polymer and composite honeycombs show evident strain rate dependence of the first maximum, or peak load. The magnitude of this dependence is process specific, but in general shows a difference of 10%–30% from the lowest to the highest strain rate. This dependence is less significant, but still present, for the effective modulus.
- Metallic honeycombs have no significant sensitivity to strain rate in regard to the effective modulus, but show some weak sensitivity to peak load.

Thus, the use of the ISO-standard recommended strain rate of 10^−3^ s^−1^ [8] is likely appropriate for metallic cellular materials, but needs careful consideration for polymeric and composite ones, since it is very probable that different results will be obtained at different strain rates. 

## Figures and Tables

**Table 1:** Summary of manufacturing processes, materials and key parameters.

Process	Equipment	Material	Layer Thickness
Fused Deposition Modeling	Stratasys 450mc	ABS-M30^TM^	250 µm
Fiber Composite Printing	MarkForged Mark Two	Onyx^TM^ and Continuous Carbon Fiber	100 µm
Electron Beam Melting	Arcam Q20	Ti6Al4V	proprietary
Laser Powder Bed Fusion	Concept Laser M2	Inconel 718	40 µm

**Table 2:** Strain rate sensitivity of the effective modulus for each of the four different processes.

Process/Material	Strain Rate Sensitivity of E* (*m*)	*R*^2^ (Quality of Fit)
FDM /ABS	0.0219	0.9535
Composite/Carbon Fiber	0.0129	0.7892
EBM/Ti6Al4V	0.0005	0.0056
LPBF/Inconel 718	0.0108	0.1303

**Table 3:** Strain rate sensitivity of, and correlations for, the peak stress for each of the four different processes.

Process/Material	Strain Rate Sensitivity of σ* (n)	*R*^2^ (Quality of Fit)
FDM /ABS	0.0405	0.9902
Composite/Carbon Fiber	0.0369	0.9561
EBM/Ti6Al4V	0.0155	0.5839
LPBF/Inconel 718	0.0042	0.5615
